# Spatial Distribution of *Echinococcus multilocularis*, Svalbard, Norway

**DOI:** 10.3201/eid1401.070565

**Published:** 2008-01

**Authors:** Eva Fuglei, Audun Stien, Nigel G. Yoccoz, Rolf A. Ims, Nina E. Eide, Pål Prestrud, Peter Deplazes, Antti Oksanen

**Affiliations:** * Norwegian Polar Institute, Tromsø, Norway; † Norwegian Institute for Nature Research, Tromsø, Norway; ‡ University of Tromsø, Tromsø, Norway; § Center for International Climate and Environmental Research, Oslo, Norway; ¶ University of Zürich, Zürich, Switzerland; # Finnish Food Safety Authority, Evira, Finland

**Keywords:** Vulpes lagopus, arctic fox, Microtus levis, sibling vole, Echinococcus multilocularis, ELISA, coproantigen, dispatch

## Abstract

In Svalbard, Norway, the only intermediate host for *Echinococcus multilocularis,* the sibling vole, has restricted spatial distribution. A survey of feces from the main host, the arctic fox, showed that only the area occupied by the intermediate host is associated with increased risk for human infection.

The cestode *Echinococcus multilocularis* is the causative agent of alveolar echinococcosis, a rare but potentially lethal human disease. In the Arctic, *E. multilocularis* depends on the arctic fox (*Vulpes lagopus,* formerly *Alopex lagopus*) or domestic dog (*Canis lupus familiaris*) as its definitive hosts, and human infections are caused by ingestion of infective eggs distributed with the feces of these hosts. A wide variety of small rodents, especially voles and lemmings of the subfamily *Arvicolinae,* can function as intermediate hosts (*1*,*2*). In 1999, *E. multilocularis* was first identified on the Arctic island Spitsbergen, in the Svalbard archipelago (*3*); since then, several human seropositive cases have been reported (*3*). These cases have caused health concerns for the public health of residents and tourists as well as for the small but flourishing tourist industry on the island.

Arctic foxes are common throughout Spitsbergen (*4*); they are most plentiful near seabird cliffs along the coast and less plentiful in the inland valleys (*5*,*6*). The only available intermediate host in Svalbard is the sibling vole (*Microtus levis,* formerly *Microtus rossiaemeridionalis*). The distribution of sibling voles on the island seems to be limited by availability of plants for food and is at present restricted to the heavily fertilized bird cliffs along the coastline in the Grumant area ([*3*], Figure). During years when vole densities were high in Grumant, voles may have spread out from the coast toward the east and west (Figure), but they have not yet established permanent populations outside the Grumant area (*3*). To evaluate how the restricted distribution of the intermediate host affects the spatial distribution of human risk for infection, we sampled fox feces at increasing distances from the core vole range (Grumant) and tested the feces for evidence of *E. multilocularis* infection by using specific *E. multilocularis* coproantigen ELISA. We also estimated the density of fox feces in Grumant and next to Longyearbyen by using line transect methods.

**Figure:**
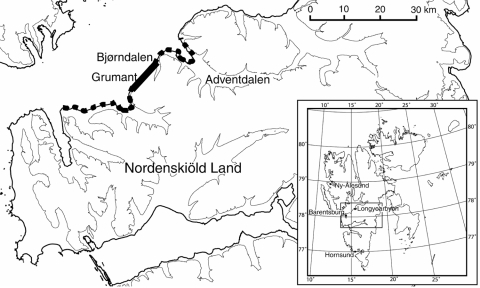
Main study area on the archipelago of Svalbard. Thick solid line, core area for sibling vole; broken line, area of distribution in peak vole year. Inset shows the 4 main settlements on the archipelago of Svalbard.

## The Study

During July 2000 and 2004, feces were collected around arctic fox dens during the annual den surveys conducted by the Norwegian Polar Institute in Svalbard. Fox feces were also collected in the Grumant area in 2004. We grouped the feces into 4 distance categories (Table, Figure): A) Grumant, the core sibling vole range, Grumant to Lille Bjørndalen; B) Bjørndalen, the neighboring valley 2–6 km from Grumant; C) Nordenskiöld Land, the more distant areas surveyed on the Nordenskiöld Land peninsula, 6–40 km from Grumant; and D) Distant, the Hornsund area 130 km south of Grumant and the Ny-Ålesund area 110 km north of Grumant. The main Norwegian settlement, Longyearbyen, (population ≈2,100) is located 12 km east of Grumant; the Russian settlement, Barentsburg, (population ≈500), is located 24 km southwest of Grumant.

Fecal samples were bagged and stored at –80 degrees for >1 week before they were processed further. Specific *E. multilocularis* coproantigen detection was performed as described by Deplazes et al. (*7*) on the samples collected in 2000; the Chekit Echino-test (Dr. Bommeli AG, Liebefeld-Bern, Switzerland) was used on the samples from 2004. As recommended by Agresti and Coull (*8*), we used score confidence limits for the proportions. The score confidence limits used are given by


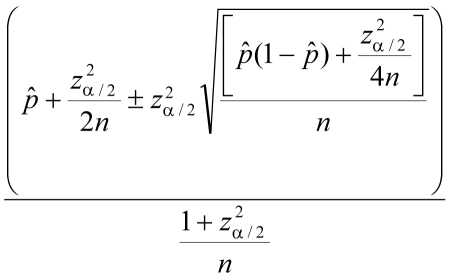


where ^^^*p* denotes the sample proportion, *n* denotes the sample size, and *z*_α/2_ denotes the 1 – α/2 quantile of the standard normal distribution (*8*); α was set to 0.05 to obtain 95% confidence limits.

We estimated the density of fox feces by using line-transect methods. Eight line transects were placed in Grumant (total length 118 m), and 10 transects of 20 m each (total length 200 m) were placed in Adventdalen, next to Longyearbyen and 15 km east of Grumant. The distance from the line to detected feces was noted; all feces detected up to 1.6 m on each side of the transect lines were included in the density estimation. The data were analyzed by using Distance 5.0 (*9*). In Adventdalen, no feces were found along the transect lines, and confidence limits for the estimated zero density of feces were calculated assuming the same detection function as in Grumant and a binomial distribution for the presence of feces along the 20-m transect lines.

The proportion of arctic fox feces that contained *E. multilocularis* showed a strong spatial pattern. The *E. multilocularis*–positive proportion within the core vole range Grumant (Table) was high, but from the nearby Bjørndalen and more distant areas on Nordenskiöld Land, no feces contained *E. multilocularis*. This finding shows that the area of high risk for human infection overlaps exactly with the core distribution range of the intermediate host. The lack of *E. multilocularis–*positive fox feces in the adjacent valley, Bjørndalen, also suggests that foxes in this area do not include part of the Grumant area in their territories. Surprisingly, fecal samples collected in the Hornsund and Ny-Ålesund areas (1 each) in 2004 were *E. multilocularis* positive. These may be false-positive results, or the feces may have come from arctic foxes that were infected in the Grumant area before a long-distance dispersal event or perhaps from even more distant locations, e.g., Northern Russia, which has been speculated to be the initial source of infection to Svalbard (*3*).

**Table:** Results of coproantigen ELISA tests of arctic fox feces for Echinococcus multilocularis*

Area	Distance, km†	2000					2004			
		n	No. positive	Proportion positive	95% CI		n	No. positive	Proportion positive	95% CI
Grumant	0	35	7	0.20	0.10–0.36		224	135	0.60	0.54–0.66
Bjørndalen	2–6	13	0	0	0–0.23		9	0	0	0–0.30
Nordenskiöld Land	6–40	91	0	0	0–0.04		74	0	0	0–0.05
Distant	110–130	0	0	0	NA		27	2	0.07	0.02–0.23

*Samples were collected in 2000 and 2004 from Grumant to Lille Bjørndalen (Grumant), from the neighboring valley Bjørndalen (Bjørndalen), from an extended area on Nordenskiöld Land peninsula (Nordenskiöld Land); and from the Ny–Ålesund and Hornsund area (Distant). CI, confidence interval. †Approximate distance from the Grumant area.

The density of fox feces in Grumant was estimated to be 4.5 feces per 100 m^2^ (95% confidence interval 1.7–12.0). No fox feces were detected along the 10 transects in Adventdalen, but the estimated confidence intervals suggest a density of 0–0.4 feces per 100 m^2^. The density of fox feces is therefore likely to be at least 11 times higher in Grumant than in Adventdalen (4.5/0.4 = 11.25). This difference can be explained by differences in fox densities as a result of lower resource availability in the inland valley of Adventdalen compared with the high resource availability at large seabird cliffs in the Grumant area (*5*,*6*).

## Conclusions

The combination of a high proportion of *E. multilocularis–*positive feces and a high density of arctic fox feces in the Grumant area suggests that this is an area of high risk for human infection. Infected foxes have the potential to spread infective *E. multilocularis* eggs far from this area; however, our data suggest that the risk for human infection drops to low levels at a very short distance from the Grumant area. A simple approach for reducing the risk for human infection on Spitsbergen is therefore to limit human exposure to this area, e.g., minimize the use of the Grumant area for recreational and tourist purposes. Further monitoring should focus on the possible vole colonization of new areas with high fox densities, i.e., areas under large seabird cliffs, which might increase the endemic range of infection. In summary, human alveolar echinococcosis risk in Svalbard is associated with the spatially restricted sibling vole population, and efficient risk management could be achieved by limiting recreational use of the vole habitat.
